# IrTMes – a stable SABRE catalyst for the hyperpolarization of [1-^13^C]-pyruvate

**DOI:** 10.1039/d6an00328a

**Published:** 2026-06-05

**Authors:** Jakoba Wacker, Jan G. Korvink, Sören Lehmkuhl

**Affiliations:** a Institute of Microstructure Technology, Karlsruhe Institute of Technology Hermann-von-Helmholtz-Platz 1 76344 Eggenstein-Leopoldshafen Germany jakoba.wacker@kit.edu soeren.lehmkuhl@kit.edu

## Abstract

Signal amplification by reversible exchange (SABRE) can enhance nuclear magnetic resonance (NMR) signals by catalyzing the transfer of spin order from *para*-hydrogen to a substrate *via* transient coordination of both to an iridium complex. An important target substrate due to its biomedical relevance is [1-^13^C]-pyruvate. Hyperpolarizing [1-^13^C]-pyruvate in an acetone water mixture (Ace-SABRE) increases the biocompatibility of hyperpolarized [1-^13^C]-pyruvate due to easier solvent removal. However, the Ace-SABRE solvent system causes rapid deactivation of the catalyst, requiring frequent renewal of the sample. Here, we introduce a more resource-efficient Ace-SABRE catalyst IrTMes, where the iridium center is ligated with 1,4-dimesityl-3-methyl-1,2,3-triazol-5-ylidene. At a *para*-hydrogen pressure of 5.17 bar, the IrTMes catalyst exhibits 17-times longer-lasting functionality under Ace-SABRE conditions and four times under exposure to the atmosphere compared to IrIMes at room temperature. This increased stability of IrTMes allows for reproducible and repeatable SABRE experiments. IrTMes achieves a signal enhancement of around 5000 at 50% *para*-hydrogen enrichment at 1.45 T, which corresponds to a polarization level of 0.62% or around 1.9% when extrapolated for 100% *para*-hydrogen. IrTMes reaches the maximum SABRE enhancement at 20 °C, avoiding the otherwise cumbersome cooling in SABRE hyperpolarization of [1-^13^C]-pyruvate. Under the same conditions, a polarization level of 1.26% (extrapolated to around 3.8% for 100% *para*-hydrogen) can be achieved with the benchmark catalyst IrIMes.

## Abstract 2

A stable polarization transfer catalyst IrTMes for the SABRE hyperpolarization of [1-^13^C]-pyruvate at room temperature.

## Graphical abstract

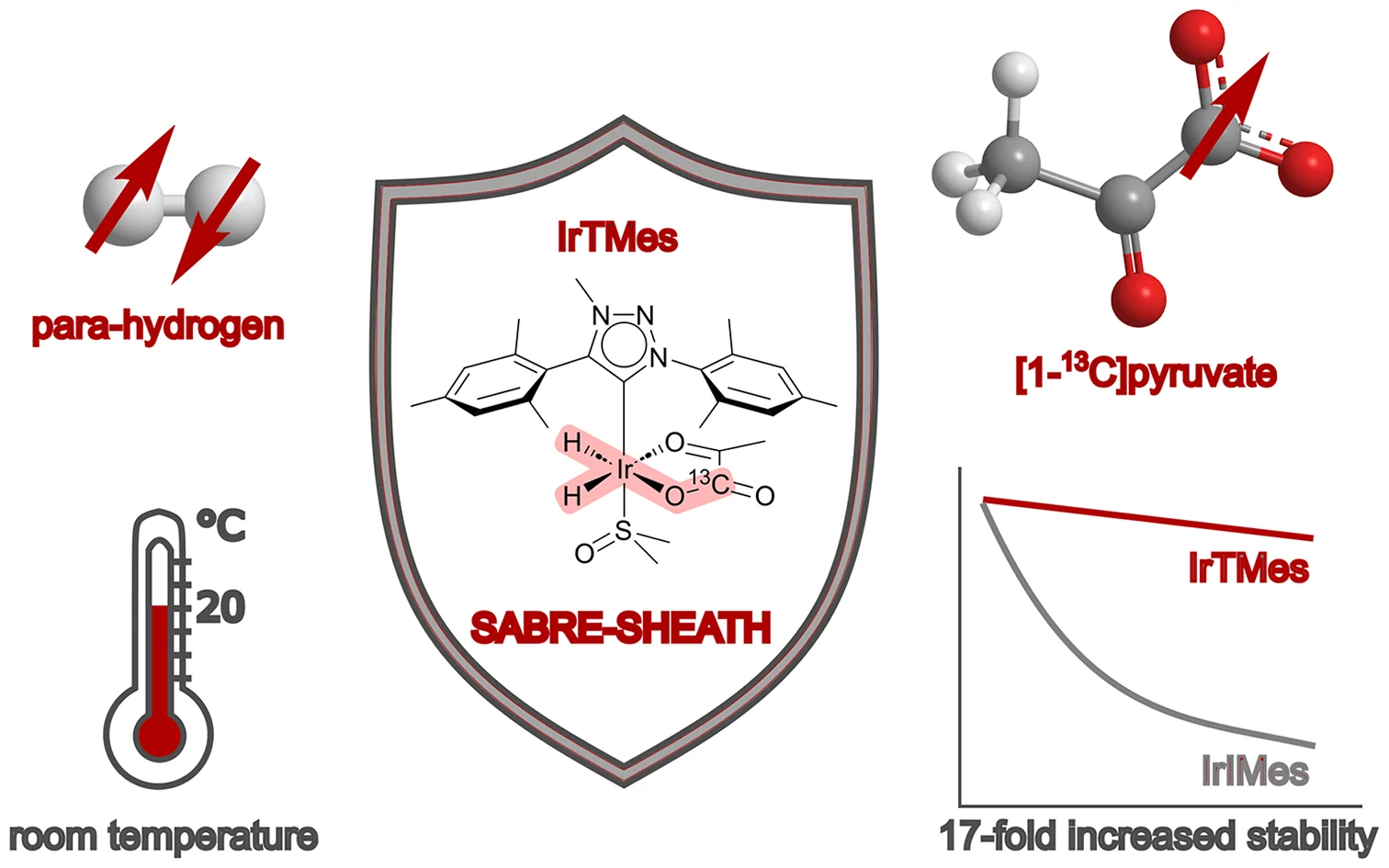

## 1 Introduction

Nuclear magnetic resonance (NMR) techniques are widespread and powerful tools in chemistry and biomedicine. However, the analytical applications of NMR are limited by low inherent sensitivity under thermal conditions due to the small population difference of the nuclear spin states. The sensitivity limitations of NMR can be overcome through hyperpolarization of the analyte.^1–3^ One technique to achieve a nonequilibrium spin order is *para*-hydrogen induced polarization (PHIP).^4^ During the catalyst-mediated process, *para*-hydrogen is added pairwise to the double or triple bond of an unsaturated organic substrate. Hyperpolarization is observed when the symmetry of the *para*-hydrogen is broken while retaining the spin correlation of the antisymmetric nuclear spin state. To hyperpolarize heteronuclei with PHIP, side-arm hydrogenation (PHIP-SAH) can be employed. In PHIP-SAH, the target molecule is functionalized with an unsaturated sidearm. After the hydrogenation with *para*-hydrogen, the side arm is removed, and the polarization is transferred to the heteronucleus *via* the spin–spin coupling network of the molecule.^5–7^

Another *para*-hydrogen-based technique is signal amplification by reversible exchange (SABRE). Unlike hydrogenative PHIP, SABRE achieves the hyperpolarization of a substrate molecule without any modifications to its chemical structure, making the process reversible and regenerative. Polarization is transferred from *para*-hydrogen to the target molecule in the presence of an iridium-based polarization transfer catalyst. Both *para*-hydrogen and the substrate bind transiently to the transition metal center, enabling the transfer of spin order through the spin–spin network of the catalyst at an appropriate polarization transfer field.^8–10^ The use of heteronuclei is particularly advantageous for *in vivo* applications due to their wide chemical shift dispersion, background-free signals and longer hyperpolarization lifetimes.^11^ For the hyperpolarization of heteronuclei such as ^13^C in [1-^13^C]-pyruvate with SABRE, the process is conducted in magnetic shields, as microtesla fields are required for spontaneous spin order transfer. This method is known as SABRE in SHield Enables Alignment Transfer to Heteronuclei (SABRE-SHEATH).^12,13^ Additionally, for the hyperpolarization of pyruvate a co-substrate such as dimethyl sulfoxide (DMSO) is required for SABRE activity.^14^

Pyruvate is a key metabolite in numerous biosynthetic pathways and therefore, a marker for various metabolic diseases.^15^ Clinical applications of [1-^13^C]-pyruvate as a molecular contrast agent hyperpolarized with dynamic nuclear polarization (DNP) enable metabolic *in vivo* imaging.^16–18^ SABRE is appealing as the polarization buildup is rapid, which is considered a challenge in applications typically using DNP. However, one requirement that must be met in order to potentially make SABRE an appropriate alternative to DNP in clinical application is the generation of biocompatible and injectable solutions.^19^

Compared to room temperature, significantly higher [1-^13^C]-pyruvate polarization levels can be achieved with SABRE when the sample is cooled during or prior to the polarization transfer. The decreased temperature slows the exchange and thereby increases the residence time of pyruvate at the catalyst, which results in a temperature optimum between 5 and 10 °C for the maximal total polarization with standard systems.^20–24^

In SABRE hyperpolarization, methanol is the most commonly used solvent. It is an acceptable compromise between the solubility of the IrIMes catalyst (IMes = 1,3-bis(2,4,6-trimethylphenyl)imidazol-2-ylidene) and the solubility of the hydrophilic sodium pyruvate. However, the high toxicity of methanol makes it unsuitable for *in vivo* applications. Although methanol can be removed by solvent evaporation, the removal process is challenging, and residual methanol is unavoidable.^25,26^ Efforts have been made to develop fully water-compatible catalysts for SABRE. Different chemical structures were proposed that showed increased solubility in aqueous solutions, but compared to IrIMes, those catalyst systems are limited by a significantly reduced SABRE efficiency.^27–30^ The transition to fully biocompatible SABRE in aqueous solution is further restricted by the low solubility of hydrogen in water. Hence, water-soluble catalysts for SABRE hyperpolarization currently are no alternative to IrIMes, especially since they have not been demonstrated in pyruvate hyperpolarization. To obtain hyperpolarized [1-^13^C]-pyruvate in water, SABRE in methanol can be followed up by a workup routine involving precipitation with ethyl acetate and reconstitution in an aqueous solution.^31^ When combined with a hydrophobic perfluorinated iridium catalyst, residual iridium levels in the aqueous solution can be drastically reduced. However, the presence of residual methanol remains unavoidable.^32^ Therefore, a different strategy is the use of an alternative solvent system that both significantly reduces the toxicity of the system and makes the solvent removal process more efficient while maintaining suitable solubility characteristics.^24^

Recently, it was shown that mixtures of acetone and water are appropriate solvent systems to meet these requirements and have been successfully applied in both [1-^13^C]-pyruvate and [2-^13^C]-pyruvate hyperpolarization.^24,33^ This so-called Ace-SABRE can not only be used to generate biocompatible aqueous solutions of hyperpolarized [1-^13^C]-pyruvate by applying a simple workup routine, but is also associated with exceptionally high levels of ^13^C polarization when applying finely tuned and optimized spin-lock-induced crossing (SLIC), a pulsed SABRE technique.^24^

While Ace-SABRE is a promising alternative to SABRE in methanol, it is associated with rapid catalyst deactivation. Although the polarization transfer catalyst is stabilized by the DMSO ligand,^14,33,34^ deactivation processes still limit its efficiency. Catalyst degradation constrains the reproducibility when acquiring a series of measurements with the same sample and also stands in contrast with efforts to make the SABRE catalyst recyclable.^26,35^ Here we present IrTMes as an alternative homogeneous catalyst with significantly increased stability. The increased stability refers to a longer-lasting functionality of the catalyst in solution. IrTMes allows for comparable measurements over extended times using the same sample and for efficient measurements at room temperature.

## 2 Results and discussion

### 2.1 Catalyst design and synthesis

Imidazol-2-ylidene-based N-heterocyclic carbenes (NHCs) are associated with robust ligand–metal bonds due to their strong σ-donor abilities as well as their steric protection through the *N*-substituents. This stabilizes the complex and catalytic intermediates.^36^ However, it is reported that the activated IrIMes catalyst exhibits a limited lifetime due to deactivating side reactions and decomposition.^37–40^ The electronic and steric properties of the carbene ligand in SABRE catalysts crucially influence the efficiency of polarization transfer. Manipulations to the *N*-substituents generally lead to adverse effects on the signal enhancement.^41^ Therefore, in the following, an alternative SABRE catalyst structure in which the *N*-substituents remain unchanged is proposed. However, the structural motif of the N-heterocyclic carbene itself is altered to a 1,2,3-triazolylidene-type mesoionic carbene to increase the stability of the compound while retaining decent efficiency.

**Scheme 1:**
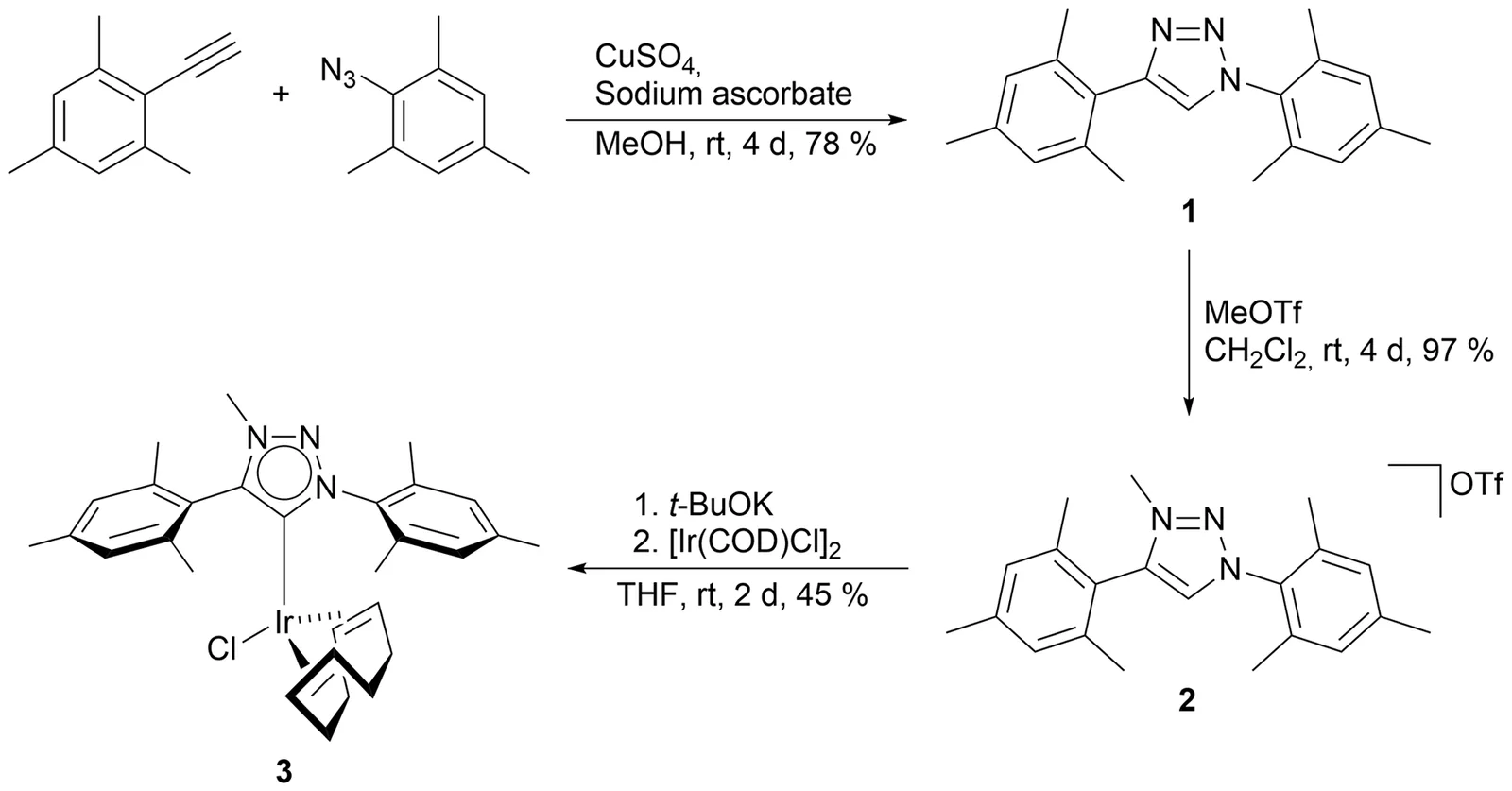
Synthetic route of the IrTMes SABRE pre-catalyst (3).

1,2,3-Triazol-5-ylidenes are associated with stronger donor properties compared to classical NHCs and form a compound class called mesoionic carbenes (MICs) because a localized structure of those compounds can only be formulated with charge separation.^42^ The 1,2,3-triazol-5-ylidene ligand, which is investigated in this study, was synthesized as an *in situ* precursor of the corresponding IrTMes (TMes = 1,4-bis(2,4,6-trimethylphenyl)1,2,3-triazol-5-ylidene) catalyst, following the synthetic route depicted in Scheme 1 (see SI for detailed synthetic information).

1,4-Dimesityl-1,2,3-triazole (1) was synthesized under click conditions *via* a copper-catalyzed azide–alkyne cycloaddition (CuAAC) from mesityl azide and mesityl acetylene.^43^ To increase the acidity of the proton in the 5-position the corresponding triazolium salt 2 was formed through *N*-3-methylation of 1 with methyl triflate.^44^ Due to the low stability of the free carbene in 1,2,3-triazol-5-ylidenes,^42^ the triazolylidene was generated *in situ* through deprotonation of the triazolium salt 2 with potassium *tert*-butoxide. By subsequent addition of [Ir(COD)Cl]_2_, the bench-stable pre-catalyst 3 was formed.^45^

### 2.2 SABRE experiments

**Fig. 1:**
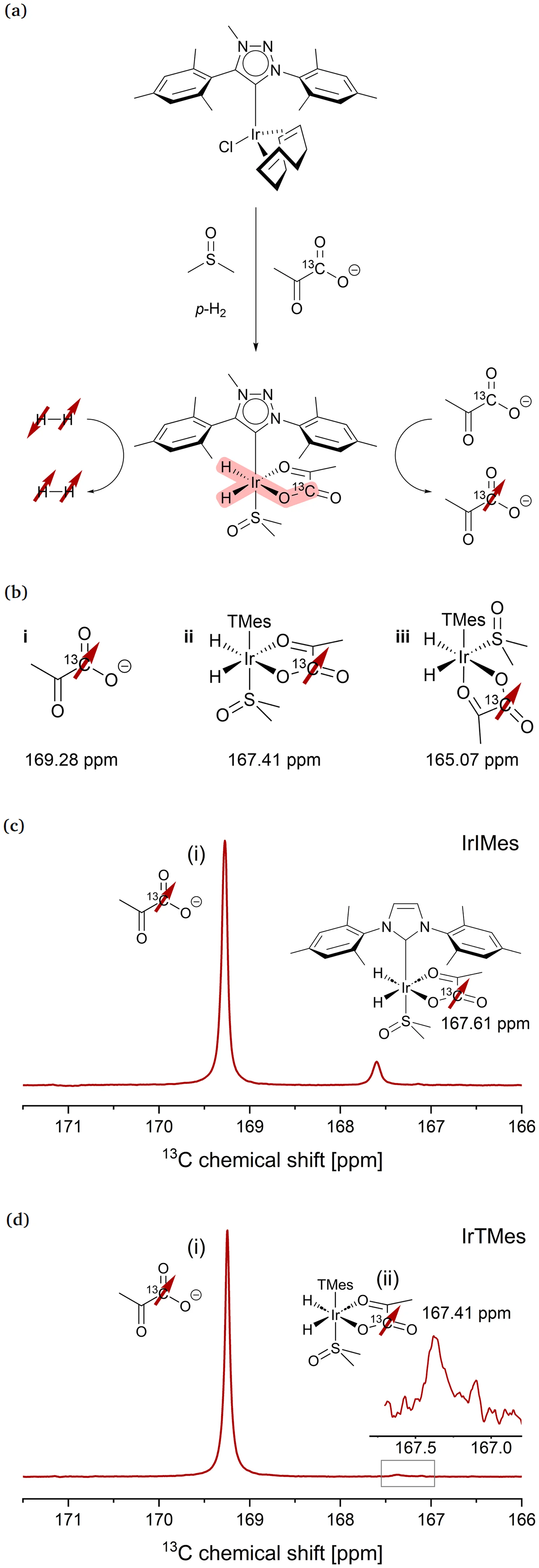
(a) Scheme of IrTMes SABRE catalyst activation and hyperpolarization process of [1-^13^C]-pyruvate. (b) Forms of hyperpolarized [1-^13^C]-pyruvate; free (i), two different bound regioisomers (ii and iii). (c and d) NMR spectra of [1-^13^C]-pyruvate hyperpolarized at room temperature with IrTMes and IrIMes. Samples consisted of 6 mM catalyst, 30 mM [1-^13^C]-pyruvate and 20 mM DMSO in an 80/20 acetone/water solution. The sample was polarized by bubbling with 50% *para*-hydrogen for 60 s at a field of −0.5 μT. Spectra were acquired at a detection field of 1.45 T.

For the SABRE hyperpolarization experiments, the pre-catalyst 3 was activated by bubbling *para*-hydrogen through a solution containing 3 (6 mM), [1-^13^C]-pyruvate (30 mM) and DMSO (20 mM) in an 80/20 acetone/water solution. Prior to data acquisition, the sample was bubbled with 50% enriched *para*-hydrogen for 60 s at a polarization transfer field of −0.5 μT inside a magnetic shield. The sample was transferred to the benchtop NMR spectrometer using an automated SABRE workstation.^46^ For detailed experimental procedure, see SI. In the following, the activated catalyst is referred to as IrTMes. The activation mechanism of the pre-catalyst and the exchange process of [1-^13^C]-pyruvate and *para*-hydrogen at the metal center are depicted in Fig. 1(a).

#### 2.2.1 Catalyst stability

**Fig. 2:**
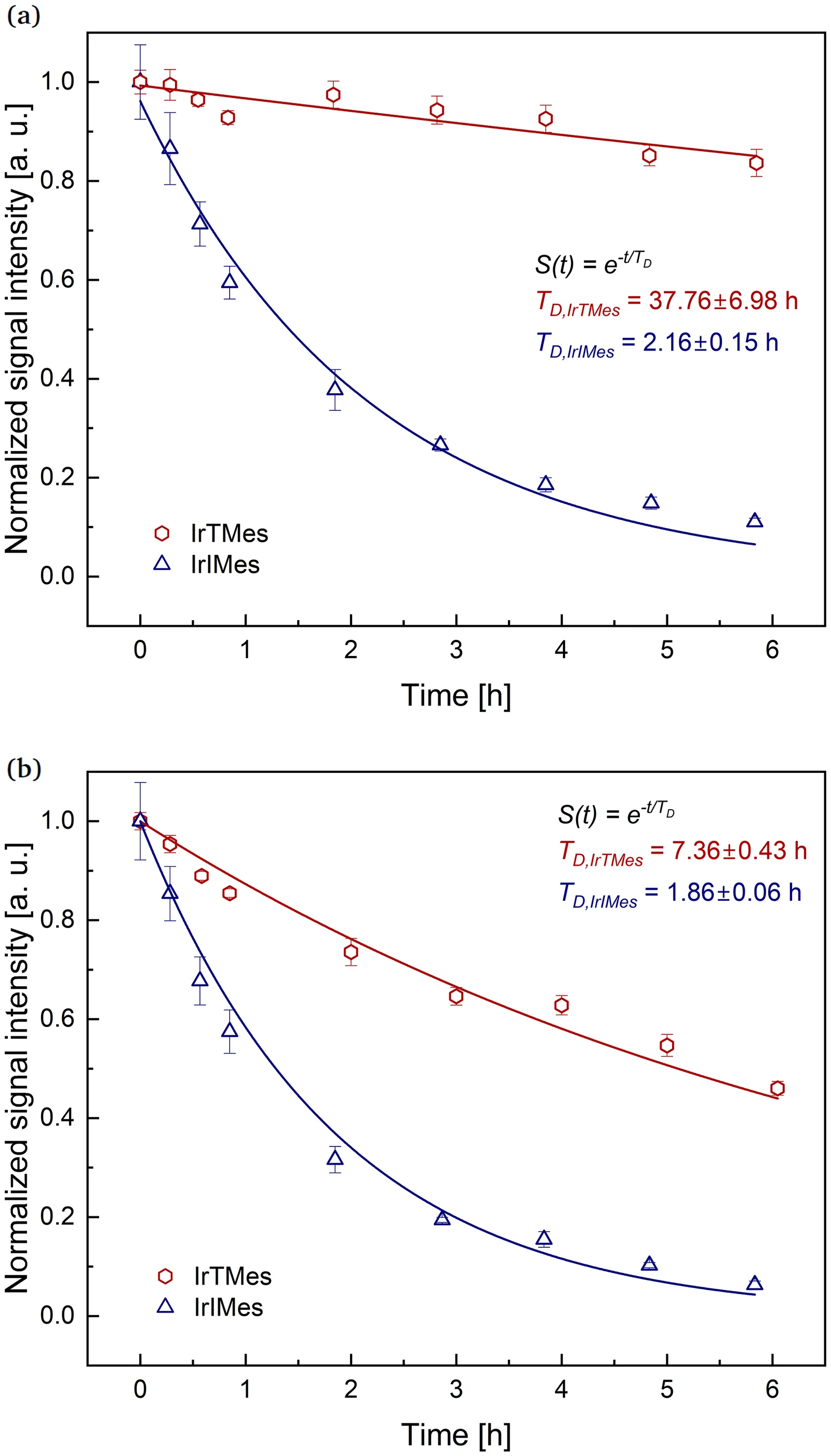
Catalyst degradation of IrIMes and IrTMes (a) under hydrogen atmosphere and (b) under ambient atmosphere as a function of time. ^13^C SABRE-SHEATH signal intensities of free [1-^13^C]-pyruvate were normalized and fitted with a mono-exponential decay function. For (a) this results in decay times of *T*_D,IrIMes_ = 2.16 h and *T*_D,IrTMes_ = 37.76 h and for (b) in *T*_D,IrIMes_ = 1.86 h and *T*_D,IrTMes_ = 7.36 h for IrIMes and IrTMes, respectively. Samples consisted of 6 mM catalyst, 30 mM [1-^13^C]-pyruvate and 20 mM DMSO in an 80/20 acetone/water solution. The sample was polarized by bubbling with 50% *para*-hydrogen for 60 s at a field of −0.5 μT. NMR spectra were detected at 1.45 T.

Although the durability of IrIMes can be increased by the addition of DMSO,^33^ catalyst degradation processes are indicated qualitatively by the observation of a color change from pale yellow to purple-brown within a few hours after activation when the sample was exposed to the atmosphere. For IrTMes, an equivalent observation was not made, even after multiple days. To quantify the rate of decomposition of activated IrIMes and investigate whether activated IrTMes actually exhibit increased durability, the signal intensities of [1-^13^C]-pyruvate achieved with both catalysts were measured in relation to time. For this, the signal intensity was measured immediately after initial catalyst activation and subsequently at 17 min intervals during the first hour, followed by hourly measurements for a total of six hours using the same sample (Fig. 2(a)). In between measurements, the sample was kept under *para*-hydrogen atmosphere. The polarization transfer was induced by bubbling with *para*-hydrogen for 60 s (including the time increments between data points) at a transfer field of −0.5 μT prior to the shuttling of the sample to the detection field and data acquisition. Each data point was averaged over three subsequent measurements and the normalized data were fitted to a mono-exponential decay function. The determined deactivation rates were 2.16 h for IrIMes and 37.76 h for IrTMes, respectively.

In addition, the effect of depressurization and atmospheric oxygen on the deactivation rate was investigated. Therefore, the series of measurements was repeated under the same conditions however, in between each measurement, the overpressure from the tube was released and the sample was opened up to the atmosphere. Before the next measurement was taken, the sample was pressurized again by bubbling 5 min with *para*-hydrogen (included in time increments between data points). Fitting the normalized data to a mono-exponential decay function resulted in deactivation rates of 1.86 h for IrIMes and 7.36 h for IrTMes, respectively (Fig. 2(b)).

Comparing the decay rates of IrTMes and IrIMes under hydrogen and ambient atmosphere shows that IrTMes exhibits a 17-fold longer-lasting functionality under hydrogen atmosphere and a fourfold longer-lasting functionality under exposure to air than IrIMes. The decay rate of IrIMes only changes insignificantly under exposure to atmospheric oxygen and hence indicates to be dominated by decomposition mechanisms in solution. For IrTMes, the decay is accelerated by a factor of five under exposure to atmospheric oxygen. One decomposition mechanism associated with activated IrIMes is the bridged dimerization of two iridium centers.^37^ Likely, this decomposition pathway is less favorable for IrTMes because of the even stronger σ-donor abilities and weaker π-electron accepting properties of the 1,2,3-triazol-5-ylidene compared to the corresponding imidazol-2-ylidene.^47^ The exceptional σ-donor strength of the TMes ligand enhances the electron density at the metal center and thereby stabilizes coordinative unsaturations, which is crucial in the prevention of formation of the bimetallic decomposition products.^48^ Likely, the increased electron density at the metal center, however, also facilitates oxidative addition mechanisms with oxygen.

#### 2.2.2 Temperature

At flow rates of 15 sccm and a pressure of 5.17 bar, SABRE hyperpolarization of [1-^13^C]-pyruvate with IrTMes shows almost exclusively free pyruvate in solution. This could also be explained by electronic stabilization of vacant coordination sites through the strong σ-donor abilities of the TMes ligand as well as increased exchange rates due to higher electron density at the metal center. In contrast, under the same conditions, hyperpolarization with IrIMes leads to a mixture of approximately 88% free and 12% catalyst-bound hyperpolarized pyruvate (Fig. 1(c and d)). At an increased pressure and flow rate of 6.89 bar and 108 sccm, respectively, the catalyst bound species (ii) and (iii) (Fig. 1(b)) are detectable for IrTMes (Fig. S1). The kinetics of the ligand exchange as well as the distribution of free and bound pyruvate strongly depend on the sample temperature. For IrIMes, low temperatures reduce the efficiency of the pyruvate exchange, leading to more bound pyruvate. Elevated temperatures increase the exchange rate. However, too rapid hydrogen exchange prevents a long enough duration at the catalyst necessary for efficient polarization transfer and ultimately limits polarization buildup on [1-^13^C]-pyruvate. This results in a temperature optimum between 5 and 10 °C for maximal total polarization with IrIMes.^20,21,23,24^

**Fig. 3:**
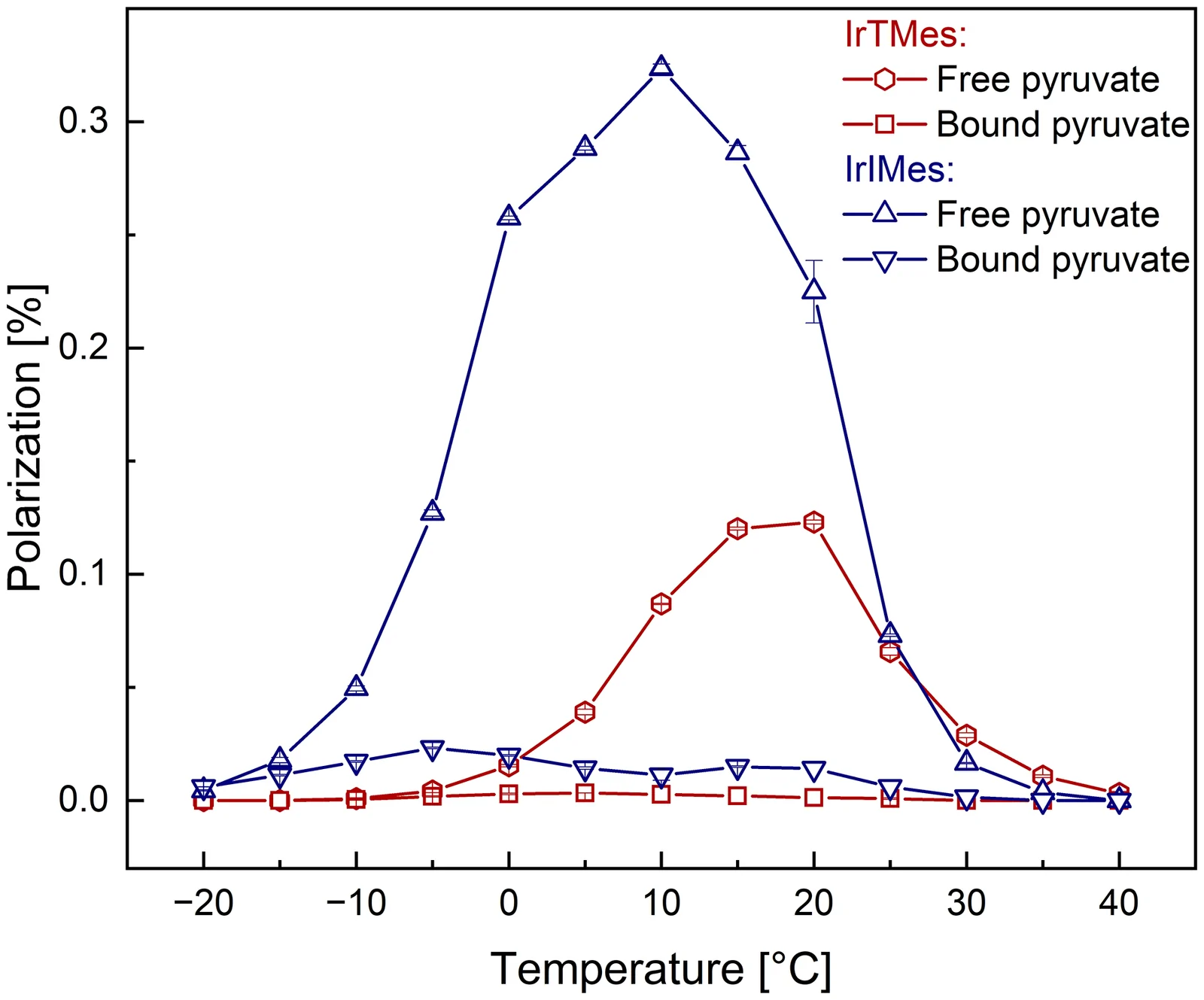
Temperature dependence of [1-^13^C]-pyruvate SABRE hyperpolarization with IrTMes and IrIMes. ^13^C SABRE-SHEATH signal intensity of free (i) and bound (ii) [1-^13^C]-pyruvate are depicted. All experiments were performed with the same sample consisting of 6 mM catalyst, 30 mM [1-^13^C]-pyruvate and 20 mM DMSO in an 80/20 acetone/water solution. The sample was polarized by bubbling with 50% *para*-hydrogen for 60 s at a field of −0.5 μT. NMR spectra were detected at 1.45 T.

To determine the temperature optimum for the SABRE-SHEATH hyperpolarization with IrTMes and the distribution of free and bound pyruvate, ^13^C signal intensities were measured as a function of temperature. The temperature was varied over a range of 60 °C in 5 °C increments (Fig. 3) during the SABRE-SHEATH polarization at a flow rate of 15 sccm and a pressure of 5.17 bar. Signal amplification was observed at temperatures above −10 °C and below 40 °C. Efficient ligand dissociation can be assumed at all temperatures within that range, since even at low temperatures, predominantly the free species is observed. At temperatures above −5 °C, free pyruvate is observed virtually exclusively. The most hyperpolarized [1-^13^C]-pyruvate was built up at 20 °C. With the optimum being at room temperature, additional temperature control becomes redundant when working with IrTMes, which facilitates experimental requirements.

Optimizing the conditions for the SABRE experiments at room temperature by increasing the pressure and flow rate to 6.89 bar and 108 sccm increases the polarization by a factor of approximately five compared to the polarizations depicted in Fig. 3. Under the optimized conditions with IrTMes, we reach signal enhancements of up to 5000-fold at room temperature with 50% *para*-hydrogen, corresponding to a polarization of 0.62%. In comparison, under the same conditions, IrIMes yielded a signal enhancement of approximately 10 000-fold, corresponding to 1.26% polarization. When extrapolated for 100% *para*-hydrogen under the same conditions, a polarization level of around 1.9% is estimated for IrTMes and 3.8% for IrIMes. While room temperature is optimal for IrTMes, lower temperatures can further increase the achievable polarization with IrIMes.

#### 2.2.3 Polarization transfer field

**Fig. 4:**
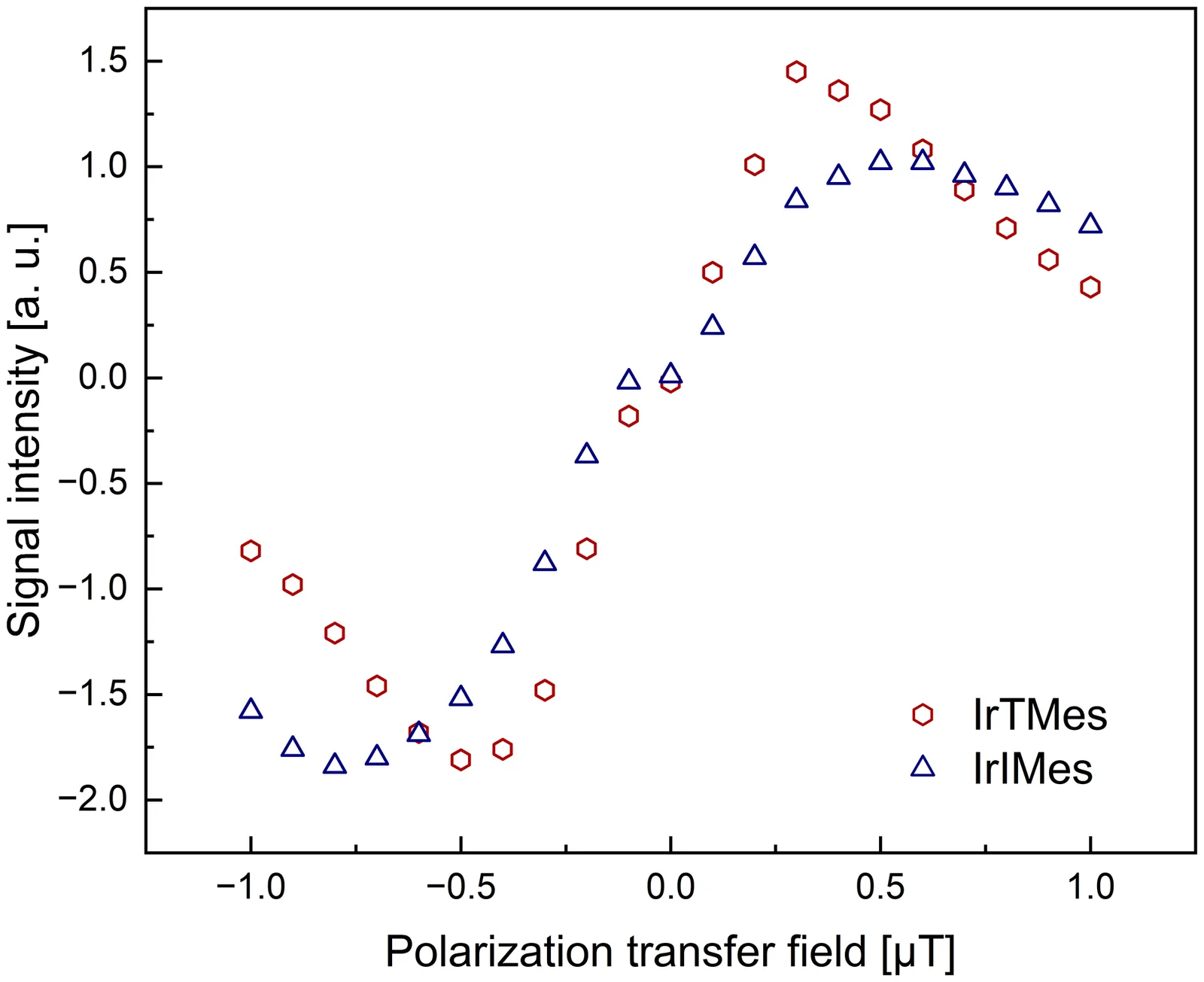
^13^C SABRE-SHEATH signal intensity of free [1-^13^C]-pyruvate as a function of the polarization transfer field. At negative transfer fields, the field's *z*-component is parallel to that of Earth's field. All experiments were performed with the same sample consisting of 6 mM catalyst, 30 mM [1-^13^C]-pyruvate and 20 mM DMSO in an 80/20 acetone/water solution. The sample was polarized by bubbling with 50% *para*-hydrogen for 60 s. NMR spectra were detected at 1.45 T.

The ideal polarization transfer field for IrTMes was determined by systematically varying the transfer field in increments of 0.1 μT from −1 μT to 1 μT (Fig. 4). The maximum signal enhancement is observed at a transfer field of −0.5 μT. The ideal polarization transfer field of IrIMes was determined to be −0.7 μT. The difference of 0.2 μT in the ideal polarization transfer field between IrIMes and IrTMes possibly results from differences in the electron density at the metal center of both catalysts. For both IrIMes and IrTMes field sweeps, the data point distribution is not perfectly symmetrical around the center, likely due to a residual field inside the shield.^49^ Positive polarization transfer fields exhibit a *z*-component inverted relative to that of Earth's magnetic field, leading to lower signal intensities. This common observation presumably results from intramolecular spin–spin interactions between the hyperpolarized ^13^C spin and neighboring spins, as the sample crosses the zero field. The inversion of the surrounding field's *z*-orientation occurs when the sample is shuttled from the magnetic shield through the Earth's field to the benchtop spectrometer, located adjacent to the shield.^46,50^

#### 2.2.4 Polarization buildup and relaxation

**Fig. 5:**
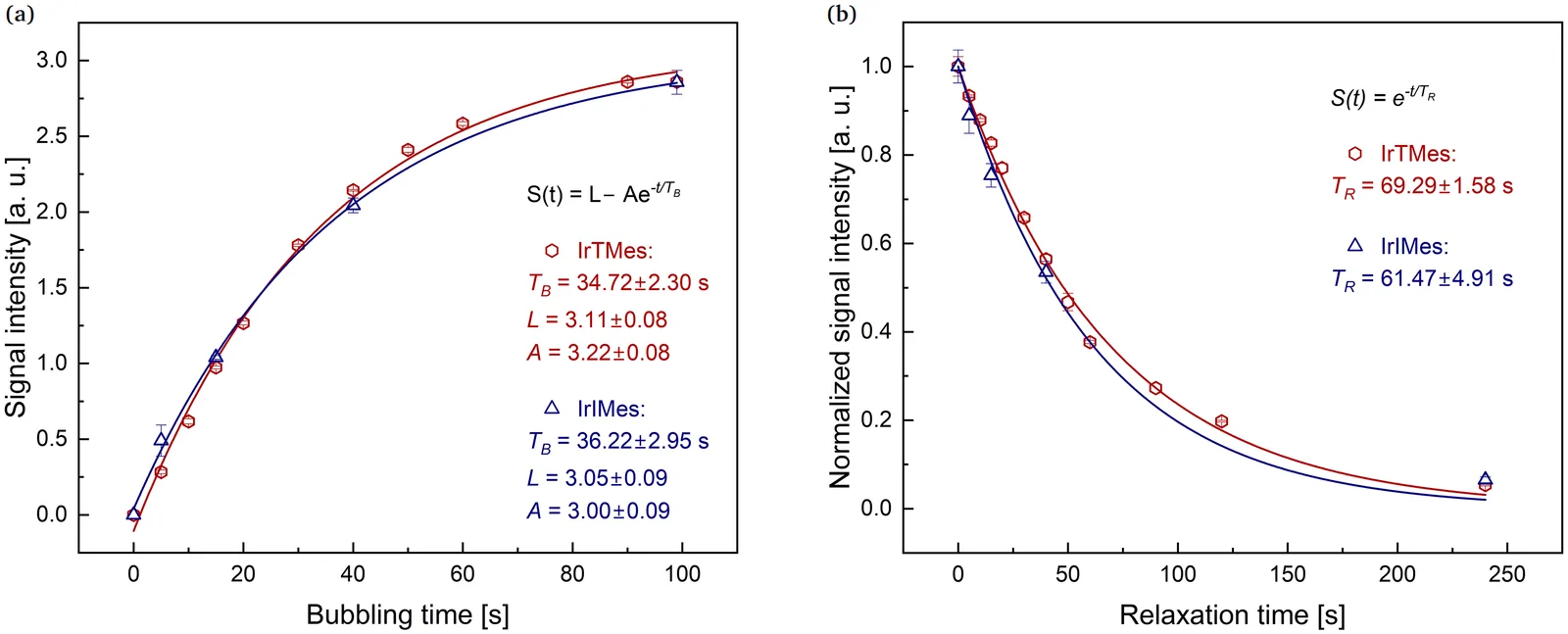
^13^C SABRE-SHEATH polarization buildup and relaxation of free [1-^13^C]-pyruvate with IrTMes and IrIMes at room temperature. (a) ^13^C signal intensity as a function of bubbling time at a magnetic field of 0.5 μT; fitted with a limited exponential growth curve; *T*_B,IrTMes_ = 35 s, *T*_B,IrIMes_ = 36 s. (b) Normalized ^13^C signal intensity as a function of duration at a magnetic field of 1.45 T after bubbling and sample shuttling; fitted with a mono-exponential decay function;*T*_R,IrTMes_ = 69 s, *T*_R,IrIMes_ = 61 s. Samples consisted of 6 mM IrTMes, 30 mM [1-^13^C]-pyruvate and 20 mM DMSO in an 80/20 acetone/water solution. The sample was polarized by bubbling with 50% *para*-hydrogen for 60 s at a field of −0.5 μT. NMR spectra were detected at 1.45 T.

The polarization buildup times of [1-^13^C]-pyruvate in solution with IrTMes and IrIMes were determined by varying bubbling times and fitting the data with limited exponential growth curves (Fig. 5(a)). For both catalysts, the data were acquired with a single sample. For IrIMes, the data were normalized and the catalyst decomposition was accounted for during post-processing by dividing the data by the degradation function prior to fitting. The buildup time *T*_B_ of 35 s for free pyruvate achieved with IrTMes is slightly faster than the buildup time of 36 s achieved with IrIMes and both are similar to that reported for free pyruvate with IrIMes in methanol at room temperature.^46^ The relaxation rate for hyperpolarized pyruvate was determined by varying durations at 1.45 T after bubbling and sample shuttling. Fitting the data with a mono-exponential decay function resulted in a relaxation time *T*_R_ of 69 s for IrTMes and 61 s for IrIMes after division with the degradation function (Fig. 5(b)). Therefore, hyperpolarized [1-^13^C]-pyruvate exhibits an approximately 1.5 times higher relaxation rate for both catalysts in Ace-SABRE compared to the relaxation rate reported for IrIMes in methanol at 1.45 T.^46^

## 3 Conclusions

This study presents an alternative SABRE hyperpolarization catalyst IrTMes that is easy to synthesize, shows optimum performance at room temperature and is more stable than the standard IrIMes catalyst.

IrTMes can be obtained through a few straightforward synthesis steps and hence contributes to the scope of SABRE catalysts. The proposed structure is the first catalyst with a 1,2,3-triazolylidene-type mesoionic carbene ligand that allows for SABRE hyperpolarization of [1-^13^C]-pyruvate. Easy to access through click reactions, 1,2,3-triazoles could prove as a useful building blocks for the design of future SABRE catalysts. Its structural similarity to the standard catalyst can help to retain efficiency, while variation of the carbene ligand can be a simple way to control the chemical and physical properties. Incorporation of different functional groups can fine-tune the steric and electronic properties, paving the way for tailored solutions for individual SABRE systems both in homogenous and heterogeneous SABRE catalysis.

For the SABRE hyperpolarization of [1-^13^C]-pyruvate with 50% *para*-hydrogen, polarization levels up to 0.62% can be achieved at the temperature optimum of IrTMes at room temperature, making additional cooling setups superfluous. Under the same conditions, IrIMes yields polarization levels up to 1.26%. While operating at room temperature simplifies the experimental procedure, further boosting the polarization by decreasing the temperature, as is possible with IrIMes, is not an available option for IrTMes, thereby limiting its ultimate efficiency.

In its solid state, the catalyst exhibits excellent long-term stability under ambient conditions and remains stable in acetone and water solutions, making it an ideal candidate for Ace-SABRE. While activated IrIMes loses half of its initial activity within the first hour and a half, activated IrTMes remains at over 85% of its initial activity after six hours, making it 17-fold more stable. Even when exposed to air, IrTMes exhibits great stability. While activated IrIMes loses almost half of its initial activity within one hour under exposure to ambient atmosphere, activated IrTMes remains at around 90% of its initial activity after the same time, making it fourfold more stable.

For application the high stability of the system streamlines sample handling, and the increased durability facilitates the use of a single sample across multiple measuring sequences. This diminishes chemical consumption and minimizes time-consuming steps like sample preparation and activation, ultimately making Ace-SABRE more sustainable by reducing waste and being more resource efficient. The increased stability of activated IrTMes even under atmospheric conditions additionally eliminates the need for inert atmosphere techniques, thereby massively simplifying the sample handling and making SABRE more accessible to a wider range of users. Furthermore, the significantly slower deactivation of IrTMes compared to IrIMes enables reproducible measurements over much longer periods of time using the same sample, and hence exploiting the renewable character of SABRE to its full potential.^8^ Repeatable polarization is an important aspect in parameter optimization and in applications requiring signal averaging over multiple scans, such as zero and ultralow-field (ZULF)^51,52^ or 2D NMR spectroscopy.^49,53,54^ Catalyst stability is also crucial in steady-state continuous flow SABRE experiments for the generation of continuous NMR signals.^55^ Those signals can *e.g.* be used in unique applications like radiofrequency amplification by stimulated emission of radiation (RASER) that result in high precision signals.^56,57^ Furthermore, the characteristics of IrTMes can contribute to the research area of new SABRE target molecules.^58^

One future aim is the application of SABRE hyperpolarized [1-^13^C]-pyruvate as a molecular contrast agent in magnetic resonance imaging (MRI). For *in vivo* studies, high degrees of polarization (>10%) are critical and can be achieved by increasing the catalyst and pyruvate concentrations in the SABRE solution. While for IrIMes concentrations around 10 mM can be achieved, IrTMes reaches its solubility limit at 6 mM, a typical catalyst concentration in SABRE experiments with [1-^13^C]-pyruvate. To increase the solubility of the catalyst, either the optimization of the solvent system or the catalyst itself is necessary. Catalyst modification is possible, *e.g.*, through easily altered click reactions. Synthetic variations of the 1,2,3-triazole precursor could also be an interesting perspective with high stability water-soluble catalyst solutions in mind.

## Author contributions

J. W.: conceptualization, formal analysis, investigation, visualization, writing – original draft; S. L., J. G. K.: funding acquisition, supervision, writing – review & editing.

## Conflicts of interest

There are no conflicts to declare.

## Supplementary Material

AN-151-D6AN00328A-s001

## Data Availability

NMR data for this article are available at RADAR4KIT at https://doi.org/10.35097/1e3xurwes8cza6ue. Supplementary information (SI) is available. See DOI: https://doi.org/10.1039/d6an00328a.
