# Supplementary material for: IrTMes – a stable SABRE catalyst for the hyperpolarization of [1-13C]-pyruvate
Source: Analyst. 2026 Jun 5. Online ahead of print. doi: 10.1039/d6an00328a (PMC13237822; doi:10.1039/d6an00328a)
Supplement: AN-OLF-D6AN00328A-s001 [file AN-OLF-D6AN00328A-s001.pdf]

Supplementary Information (SI) for Analyst.
This journal is © The Royal Society of Chemistry 2026

Supporting Information for

IrTMes – A stable SABRE catalyst for the hyperpolarization of [1-^{13}C]pyruvate

Jakoba Wacker,*[a] Jan G. Korvink,[a] and Sören Lehmkuhl*[a]

[a] *Institute of Microstructure Technology, Karlsruhe Institute of Technology, Hermann-von-Helmholtz-Platz 1, 76344 Eggenstein-Leopoldshafen, Germany. E-mail: jakoba.wacker@kit.edu; soeren.lehmkuhl@kit.edu*

Contents

1 Experimental procedures **1**
1.1 Sample preparation 1
1.2 Experimental setup and hyperpolarization experiments 1

2 Calculation of signal enhancement and polarization **1**

3 NMR Spectra of hyperpolarized [1-^{13}C]pyruvate **2**

4 Synthesis **3**
4.1 General synthetic procedures 3
4.1.1 Synthesis of 1,4-dimesityl-1,2,3-triazole (**1**) 3
4.1.2 Synthesis of [MesMesTrzMe]OTf (**2**) 3
4.1.3 Synthesis of IrTMes (**3**) 4
4.1.4 Synthesis of IrIMes 4

5 NMR spectra **5**

1 Experimental procedures

1.1 Sample preparation

Para-hydrogen (p-H_2, 50% enrichment) used for SABRE experiments was continuously generated by flowing hydrogen gas over Fe_2O_3 conversion catalyst at 77 K. Samples for ^{13}C SABRE-SHEATH polarization were prepared in a solvent mixture of 80% acetone-H6 and 20% D_2O. For this 6 mM precatalyst and 20 mM DMSO were dissolved under sonication in acetone. 30 mM [1-^{13}C]pyruvate was dissolved in D_2O. Both solutions were combined.

1.2 Experimental setup and hyperpolarization experiments

For hyperpolarization experiments, a 5 mm NMR tube was loaded with 700 μL of the sample suspension. The precatalyst was activated by bubbling *para*-hydrogen through the sample solution for 30 min at room temperature with an overpressure of 5.17 bar (75 psi) and a flow rate of 15 mL/min (15 sccm). The sample tube was transferred between the magnetic shield (Twinleaf MS-2) and the benchtop NMR spectrometer using the automated SABRE workstation recently published by Yang et al.[1] Prior to polarization, [1-^{13}C]pyruvate was brought to thermal equilibrium by shuttling the sample into the magnetic shield without applying a field for 30 s. Samples were polarized in the magnetic shield through *para*-hydrogen bubbling for 60 s at room temperature at an internal magnetic field of −0.5 µT. For variable-temperature experiments, the temperature was controlled by a liquid flow of pre-tempered ethanol during bubbling inside the shield. Spectroscopic measurements were started automatically after the sample tube was placed inside the NMR spectrometer. All NMR spectra were measured on a Magritek Spinsolve 60 Ultra benchtop NMR instrument operating at a magnetic field of 1.45 T. ^{13}C signals were detecetd in a single scan at a dwell time of 200 µs with 16384 points. The RF pulse length for $\frac{\pi}{2}$ excitation was 70.6 µs. NMR data was processed using MNOVA. Data were evaluated after automated baseline and phase correction. The acquired free induction decays (FIDs) were apodized with an exponential filter of 0.3 Hz.

The polarization was determined at an overpressure of 6.89 bar (100 psi) and a flow rate of 15 mL/min (108 sccm) with manual sample shuttling. All other experimental conditions remained unchanged.

2 Calculation of signal enhancement and polarization

The ^{13}C signal enhancement was calculated using a thermal reference spectrum of pure (95.4%) [1-^{13}C]vinyl acetate acquired at a 1.45 T magnetic field on the same Magritek Spinsolve 60 Ultra benchtop NMR instrument.

The concentration of the [1-^{13}C]vinyl acetate reference is calculated by the equation

$$c = 0.954 \cdot \frac{\rho}{M} = 0.954 \cdot \frac{0.934\,\frac{\text{g}}{\text{mL}}}{87.08\,\frac{\text{g}}{\text{mol}}} = 10.23 \times 10^{-3}\,\frac{\text{mol}}{\text{mL}} = 10.23\,\frac{\text{mol}}{\text{L}} \tag{1}$$

The signal enhancement $\epsilon_{^{13}C}$ is calculated from the integrated signal intensities of hyperpolarized [1-^{13}C]pyruvate S_{HP} and thermal [1-^{13}C]vinyl acetate S_{Ref}, their corresponding concentrations c_{HP} and c_{Ref} and the arias of the samples inside the 5 mm NMR tube A_{HP} and A_{Ref}

$$\epsilon_{^{13}C} = \frac{S_{HP}}{S_{Ref}} \cdot \frac{c_{Ref}}{c_{HP}} \cdot \frac{A_{Ref}}{A_{HP}} = \frac{9.32}{0.67} \cdot \frac{10.23 \times 10^{3}\,\frac{\text{mmol}}{\text{L}}}{30\,\frac{\text{mmol}}{\text{L}}} \cdot 1.053 = 4947 \tag{2}$$

The thermal ^{13}C polarization level is calculated from the gyromagnetic ratio γ, the magnetic field B_0, the reduced Planck constant $\hbar$, the Boltzmann constant k and the temperature T

$$P_{th} = \frac{\gamma B_0 \hbar}{2kT} = \frac{67.262\,\text{MHz} \cdot 1.45\,\text{T} \cdot 1.054\,571\,8 \times 10^{-34}\,\text{Js}}{2 \cdot 1.380\,649 \times 10^{-23}\,\frac{\text{J}}{\text{K}} \cdot 298\,\text{K}} = 1.25 \times 10^{-4}\% \tag{3}$$

This results in a polarization level for hyperpolarized [1-^{13}C]pyruvate of

$$P_{^{13}C,50\%} = \epsilon_{^{13}C} \cdot P_{th} = 4947 \cdot 1.25 \times 10^{-4}\% = 0.62\% \tag{4}$$

Extrapolated to 100% *para*-hydrogen enrichment that results in

$$P_{^{13}C} = 3 \cdot \epsilon_{^{13}C,50\%} = 3 \cdot 0.62\% = 1.9\% \quad (5)$$

3 NMR Spectra of hyperpolarized [1-^{13}C]pyruvate

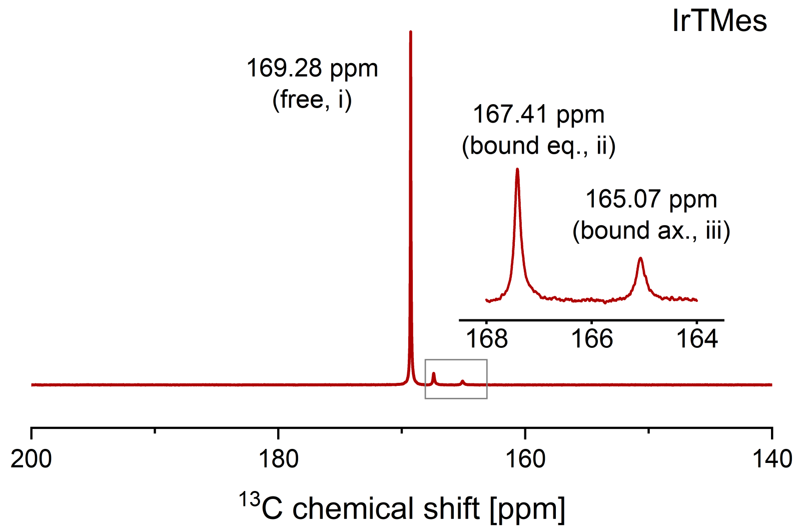

Fig. S1: NMR spectrum of [1-^{13}C]-pyruvate hyperpolarized at room temperature, 6.89 bar and 108 sccm with IrTMes. Samples consisted of 6 mM IrTMes, 30 mM [1-^{13}C]-pyruvate and 20 mM DMSO in an 80/20 acetone/water solution. The sample was polarized by bubbling with 50% *para*-hydrogen for 60 s at a field of −0.5 µT. Spectra were acquired at a detection field of 1.45 T.

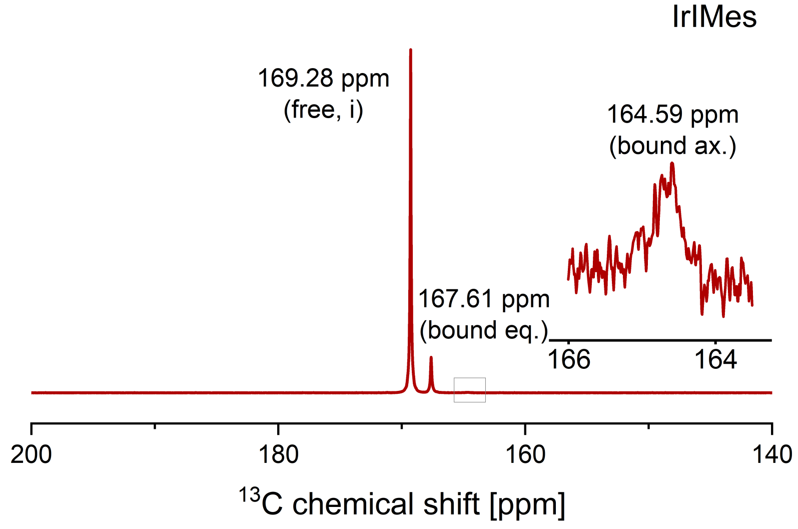

Fig. S2: NMR spectrum of [1-^{13}C]-pyruvate hyperpolarized at room temperature, 6.89 bar and 108 sccm with IrIMes. Samples consisted of 6 mM IrIMes, 30 mM [1-^{13}C]-pyruvate and 20 mM DMSO in an 80/20 acetone/water solution. The sample was polarized by bubbling with 50% *para*-hydrogen for 60 s at a field of −0.5 µT. Spectra were acquired at a detection field of 1.45 T.

4 Synthesis

4.1 General synthetic procedures

Sodium ascorbate, copper(II) sulfate and methyl trifluoromethanesulfonate were purchased from Thermo Fisher Scientific. 2,4,6-Trimethylphenyl acetylene was purchased from abcr. *Tert*-butoxide, $[Ir(cod)Cl]_2$ and 1,3-Bis(2,4,6-trimethylphenyl)-imidazolium chloride were purchased from Sigma-Aldrich. Solvents were purchased from Fisher Scientific and Sigma-Aldrich. Commercially available chemicals were used without further purification. All reactions were performed under an inert argon atmosphere using standard Schlenk techniques. 2,4,6-Trimethylphenyl azide was synthesized according to a literature procedure.[2] Solvents were degassed before use, applying standard techniques. Compounds were characterized by Nuclear Magnetic Resonance (NMR) using a Bruker Avance 400 spectrometer. For NMR spectra chloroform-d3 was used as solvent. Chemical shifts are reported in ppm, referenced to the residual solvent peaks.[3] Coupling constants are given in hertz (Hz) and assigned as singlet (s), doublet (d), triplet (t), quartet (q), quintet (quint) and multiplet (m). The mass spectrum was acquired on an Agilent 1200 HPLC 6230 TOF mass spectrometer equipped with an electrospray ionization (ESI) source.

4.1.1 Synthesis of 1,4-dimesityl-1,2,3-triazole (1)

The synthesis of **1** was derived from literature known procedure.[4]

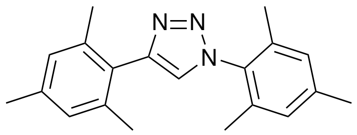

Under inert conditions 2,4,6-trimethylphenyl azide (6.2 mmol, 1 g) was suspended in methanol (20 mL). Sodium ascorbate (12 mmol, 2.38 g), copper(II) sulfate (5.6 mmol, 0.89 g) and 2,4,6-trimethylphenyl acetylene (6.8 mmol, 0.98 g, 1.1 mL) were added. The reaction mixture was stirred for 4 d. Brine (100 mL) and ethyl acetate (100 mL) were added and the solids were filtered off. The aqueous phase was extracted with ethyl acetate (3x 100 mL) and dichloromethane (3x 100 mL). The combined organic phase was washed with brine (100 mL) and dried over sodium sulfate. Volatiles were removed under reduced pressure and **1** was obtained as a brown solid with 97% yield (1.84 g).

^{1}H NMR (400 MHz, $CDCl_3$): δ = 7.54 (s, 1H, Trz-*H*), 7.03 (s, 2H, Mes-*H*), 6.98 (s, 2H, Mes-*H*), 2.38 (s, 3H, *p*-CH_3), 2.34 (s, 3H, *p*-CH_3), 2.18 (s, 6H, *o*-CH_3), 2.04 (s, 6H, *o*-CH_3) ppm.
^{13}C{^{1}H} NMR (101 MHz, $CDCl_3$): δ = 140.1, 138.4, 137.9, 135.3, 133.8, 129.2, 128.5, 128.2, 127.3, 120.8, 77.4, 21.3, 20.8, 17.4 ppm.

4.1.2 Synthesis of [MesMesTrzMe]OTf (2)

The synthesis of **2** was derived from literature known procedure.[5]

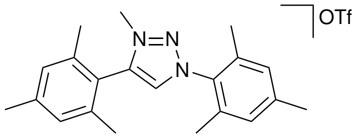

Under inert conditions **1** (1.64 mmol, 500 mg) was dissolved in dry dichloromethane (20 mL). After cooling the solution to −77 °C methyl trifluoromethanesulfonate (2.46 mmol, 404 mg, 270 µL) was added dropwise to the solution. The reaction mixture was brought to room temperature and was stirred for 4 d. The solvent was removed under reduced pressure. The brown residue was dissolved in a small amount of dichloromethane (4 mL) and precipitated by addition of diethyl ether (100 mL). The precipitant was collected by filtration, washed with cold diethyl ether (2x 50 mL) and **2** was obtained as a colorless solid with 78% yield (604 mg).

^{1}H NMR (400 MHz, $CDCl_3$): δ = 8.75 (s, 1H, Trz-*H*), 7.07 (s, 2H, Mes-*H*), 7.06 (s, 2H, Mes-*H*), 4.16 (s, 3H, Trz-CH_3), 2.38 (s, 3H, *p*-CH_3), 2.37 (s, 3H, *p*-CH_3), 2.13 (s, 6H, *o*-CH_3), 2.12 (s, 6H, *o*-CH_3) ppm.

^{13}C{^{1}H} NMR (101 MHz, $CDCl_3$): δ = 143.0, 142.9, 142.9, 138.2, 134.3, 132.2, 131.3, 130.1, 129.6, 117.2, 77.4, 38.3, 21.5, 21.4, 20.1, 17.3 ppm.

4.1.3 Synthesis of IrTMes (3)

The synthesis of **3** was derived from literature known procedure.[6]

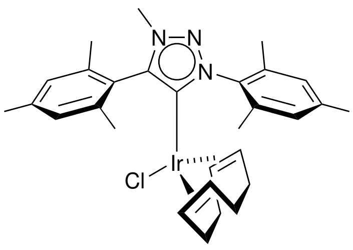

Under inert conditions **2** (0.85 mmol, 400 mg) was dissolved in dry tetrahydrofuran (40 mL) and the solution was cooled to −77 °C. Potassium *tert*-butoxide was added slowly in small portions to the solution. The solution was brought to room temperature and stirred for 15 min. $[Ir(cod)Cl]_2$ was added and the reaction mixture was stirred for 2 d. The solvent was removed under reduced pressure and the brown residue was dissolved in dichloromethane (100 mL) and filtered over a silica plug. After removal of the volatiles under reduced pressure, the solid residue was dissolved in a small amount of dichloromethane and precipitated by addition of hexane. The solvents were decanted off and **3** was obtained as a yellow solid with 45% yield (249 mg).
^{1}H NMR (400 MHz, $CDCl_3$): δ = 7.06-6.95 (m, 4H, Mes-*H*), 4.24–4.05 (m, 2H, COD-*H*), 3.75 (s, 3H, Trz-CH_3), 3.03–2.76 (m, 2H, COD-C*H*), 2.39–2.35 (m, 6H, *p*-CH_3), 2.28–2.16 (m, 12H, *o*-CH_3), 1.89–1.768 (m, 2H, COD-CH_2), 1.75–166 (m, 2H, COD-CH_2), 1.45–1.34 (m, 2H, COD-CH_2), 1.31–1.21 (m, 2H, COD-CH_2) ppm.
^{13}C{^{1}H} NMR (101 MHz, $CDCl_3$): δ = 170.9, 144.3, 139.7, 136.5, 128.9, 128.1, 124.4, 120.5, 81.0, 77.4, 51.6, 36.1, 33.7, 29.9, 29.4, 21.5, 21.4, 21.3, 18.9, 1.1 ppm.
HRMS: *m/z* $[M–Cl+H]^+$ calculated for $C_{29}H_{38}IrN_3$: 621.2695; found: 621.2692.

4.1.4 Synthesis of IrIMes

The synthesis of IrIMes was derived from literature known procedure.[7]

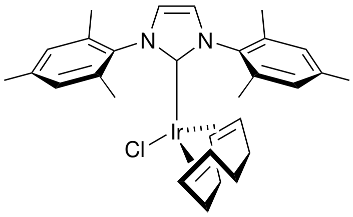

$[Ir(cod)Cl]_2$ (1.2 mmol, 806 mg) and 1,3-Bis(2,4,6-trimethylphenyl)-imidazolium chloride (2 mmol, 682 mg) were combined in a Schlenk flask and dissolved in dry THF (30 mL). A solution of potassium *tert*-butoxide (2.4 mmol, 269 mg) in dry THF (20 mL) was added dropwise to the solution under vigorous stirring. The reaction mixture was stirred at room temperature for an additional 20 min. DCM (50 mL) and brine (50 mL) were added and the organic phase was separated. The aqueous phase was extracted twice with DCM (50 mL). The combined organic phases were washed with brine (50 mL) and dried over sodium sulfate. After removing the volatiles under reduced pressure the solid residue was dissolved in a small amount of DCM and filtered over a silica plug. IrIMes was obtained as a yellow solid with 85% yield (1.089 g).

^{1}H NMR (400 MHz, $CDCl_3$): δ = 7.01 (s, 2H, Mes-*H*), 6.98 (s, 2H, Mes-*H*), 6.95 (s, 2H, Im-*H*), 4.20–4.10 (m, 2H, COD-C*H*), 3.02–2.92 (m, 2H, COD-C*H*), 2.36 (s, 6H, Mes-CH_3), 2.35 (s, 6H, Mes-CH_3), 2.16 (s, 6H, Mes-CH_3), 1.79–1.58 (m, 4H, COD-CH_2) 1.40–1.19 (m, 4H, COD-CH_2) ppm.
^{13}C{^{1}H} NMR (101 MHz, $CDCl_3$): δ = 80.9, 138.8, 137.5, 136.2, 134.5, 129.6, 128.3, 123.4, 82.7, 51.6, 33.6, 29.1, 21.3, 19.8, 18.4 ppm.

5 NMR spectra

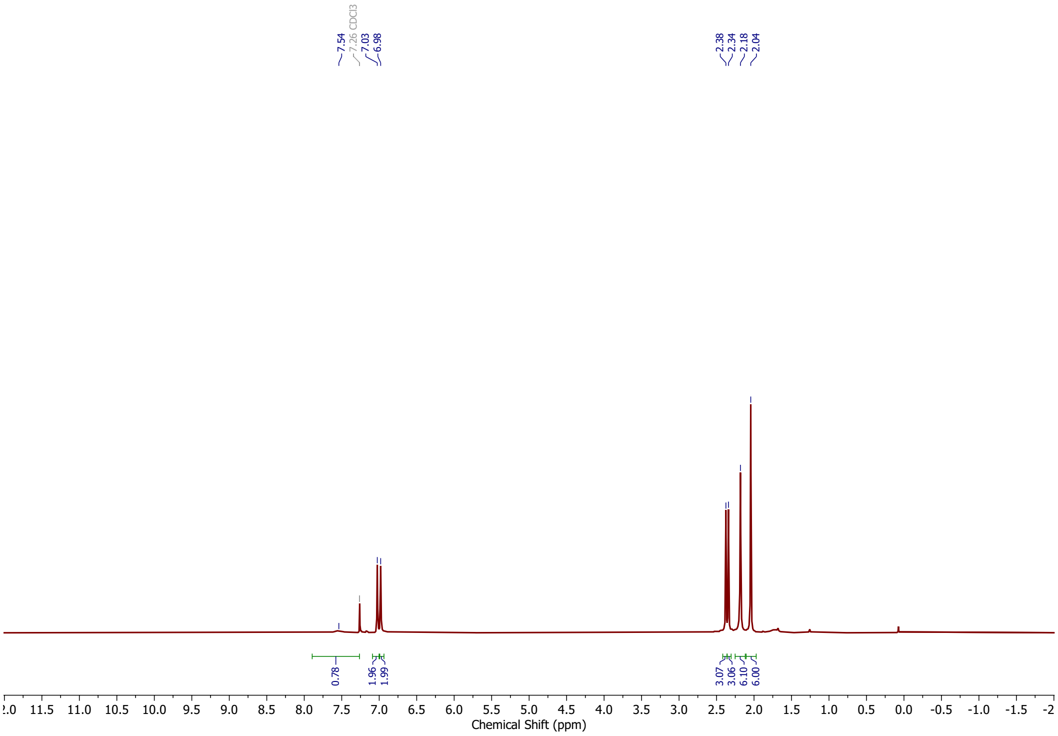

Fig. S3: ^{1}H NMR spectrum of 1,4-dimesityl-1,2,3-triazole (**1**) (400 MHz, $CDCl_3$).

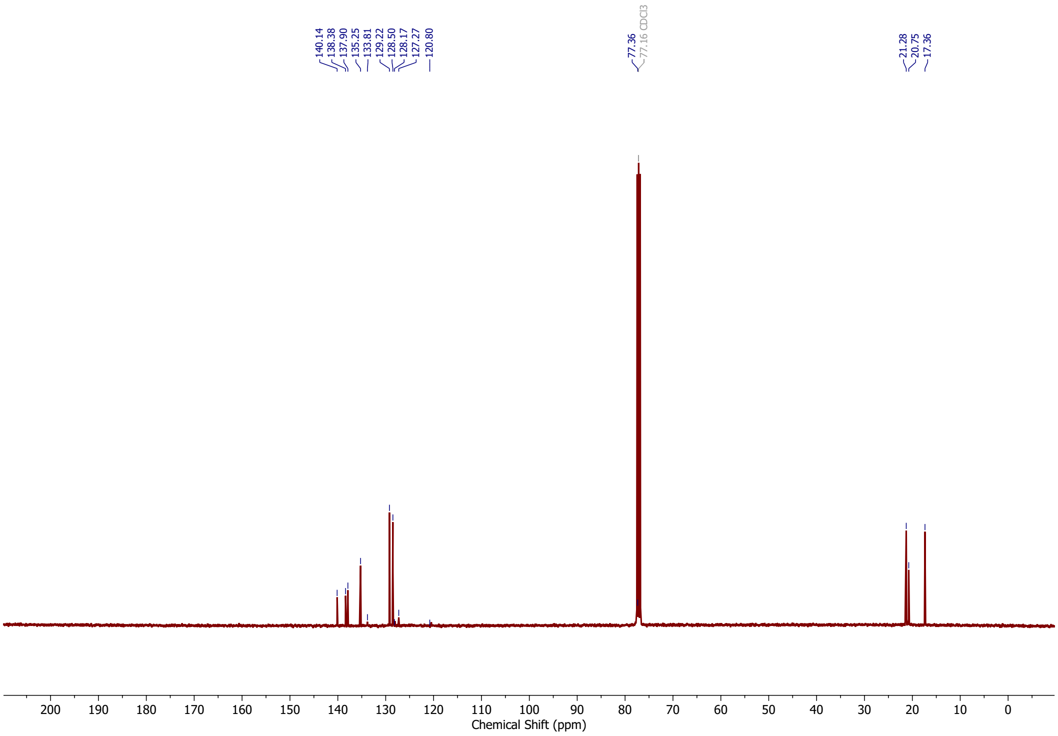

Fig. S4: $^{13}C\{^{1}H\}$ NMR spectrum of 1,4-dimesityl-1,2,3-triazole (**1**) (101 MHz, $CDCl_3$).

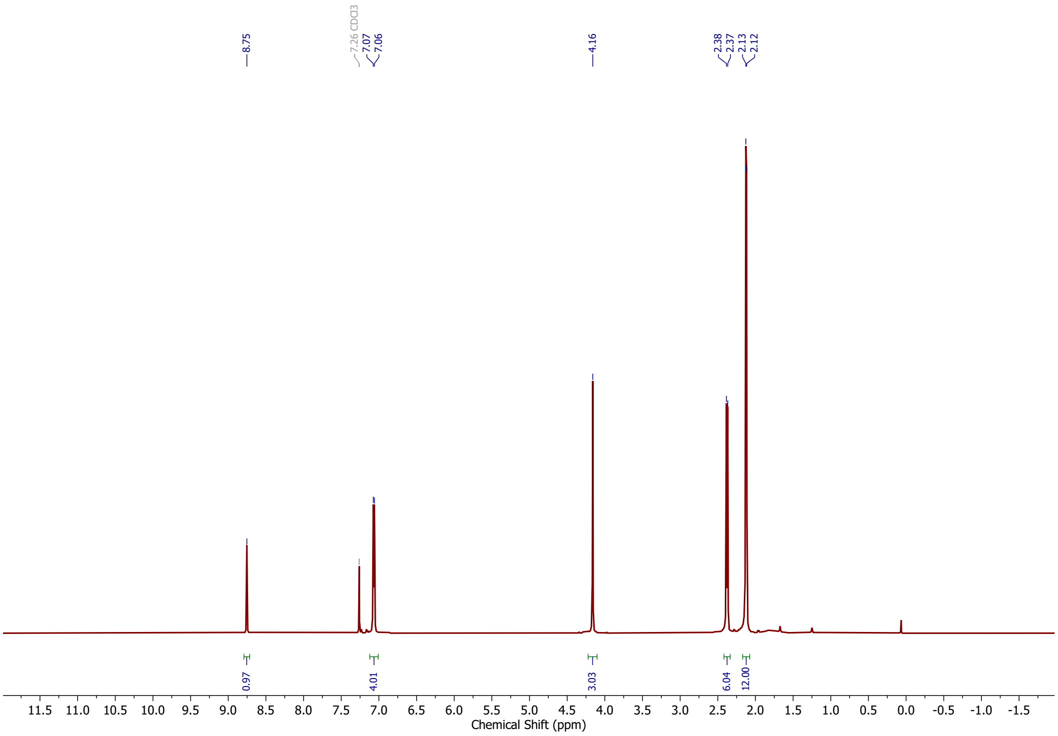

Fig. S5: ^{1}H NMR spectrum of [MesMesTrzMe]OTf (**2**) (400 MHz, $CDCl_3$).

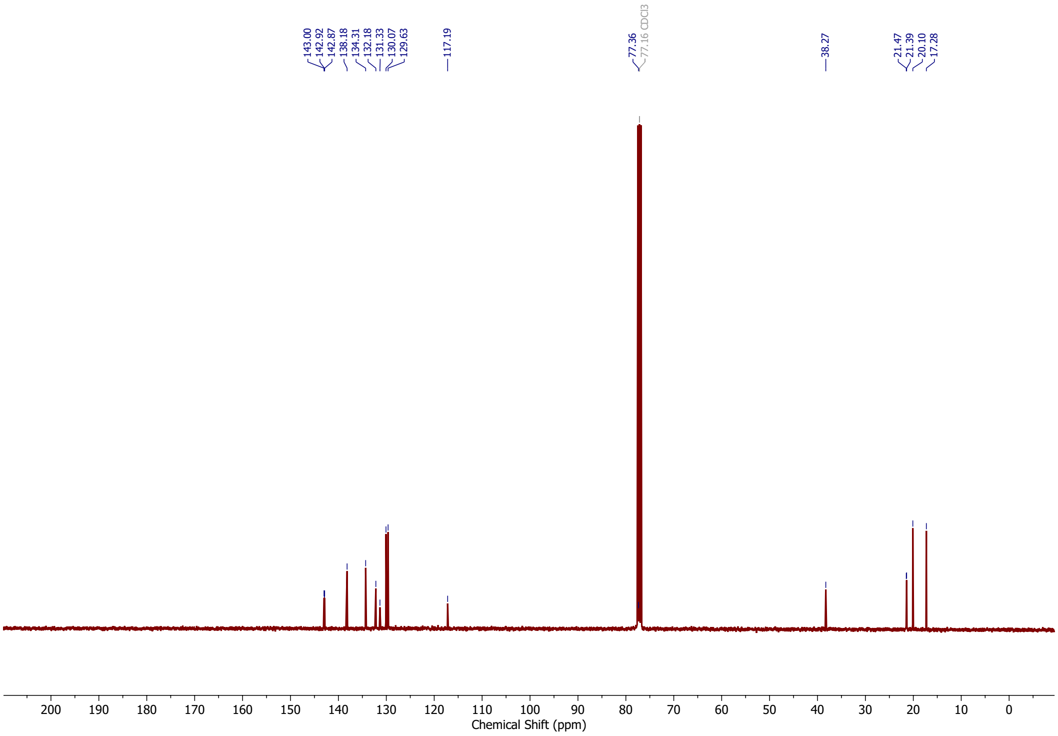

Fig. S6: ^{13}C{^{1}H} NMR spectrum of [MesMesTrzMe]OTf (**2**) (101 MHz, $CDCl_3$).

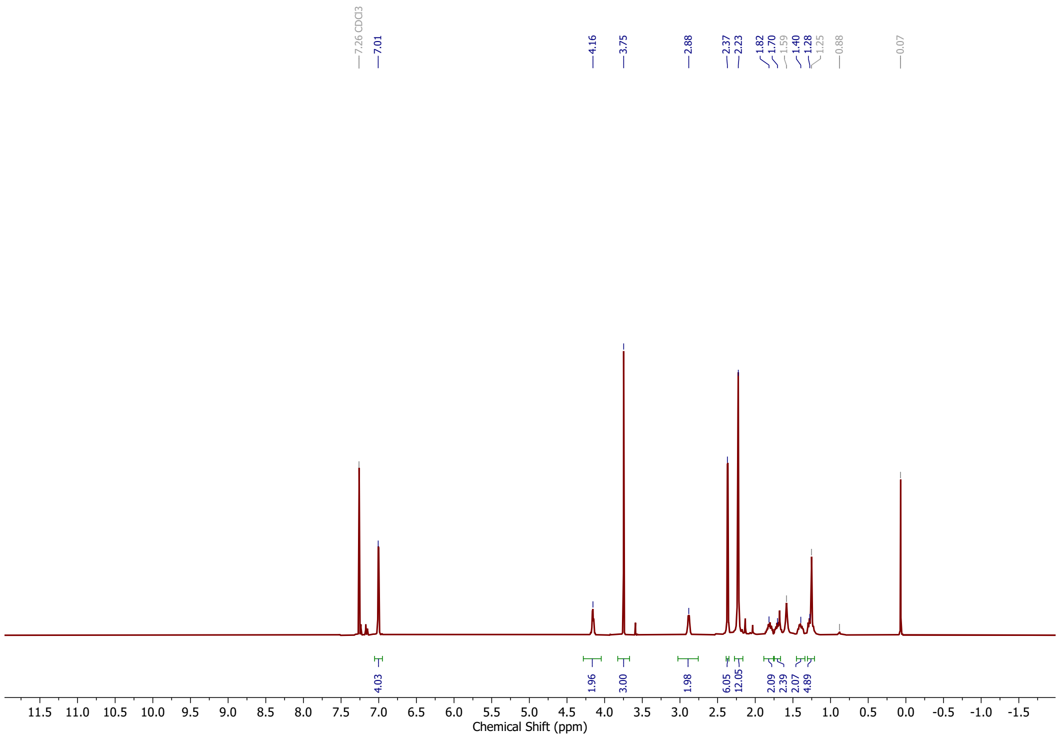

Fig. S7: ^{1}H NMR spectrum of IrTMes (**3**) (400 MHz, $CDCl_3$). Signals corresponding to residual solvent (hexane), water, and silicone grease are indicated.

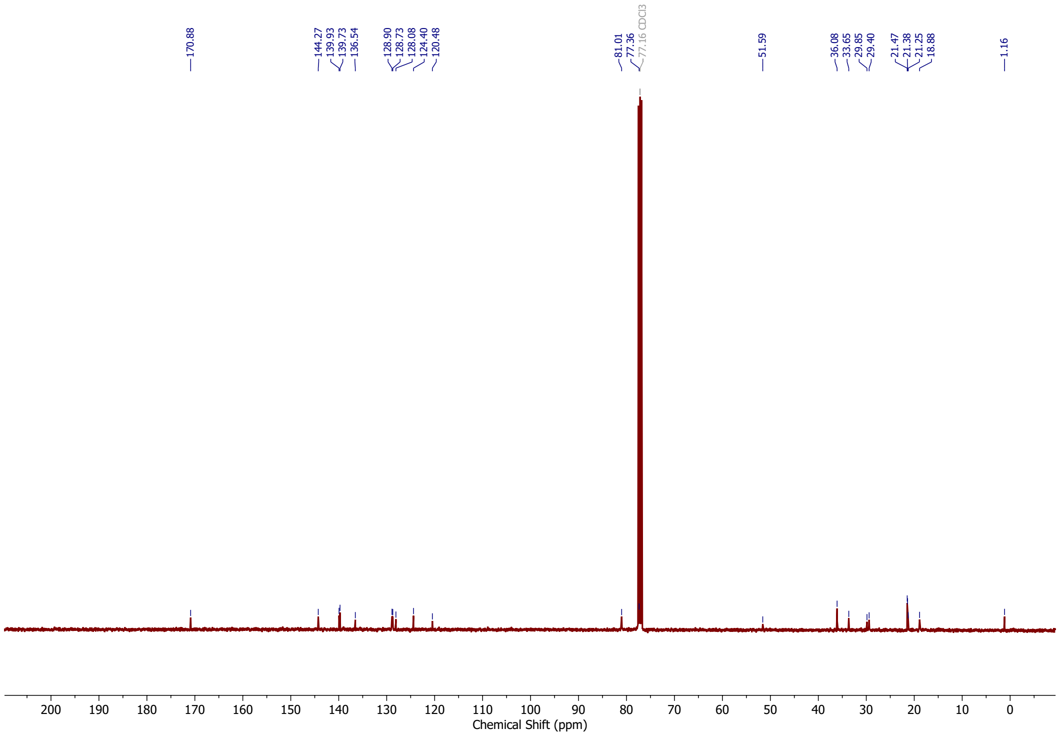

Fig. S8: ^{13}C{^{1}H} NMR spectrum of IrTMes (**3**) (101 MHz, $CDCl_3$).

References

[1] J. Yang, R. Xin, S. Lehmkuhl, J. G. Korvink and J. J. Brandner, *Sci. Rep.*, 2024, **14**, 21022.

[2] S. W. Kwok, J. R. Fotsing, R. J. Fraser, V. O. Rodionov and V. V. Fokin, *Org. Lett.*, 2010, **12**, 4217–4219.

[3] G. R. Fulmer, A. J. M. Miller, N. H. Sherden, H. E. Gottlieb, A. Nudelman, B. M. Stoltz, J. E. Bercaw and K. I. Goldberg, *Organometallics*, 2010, **29**, 2176–2179.

[4] J. Liebscher and S. Khan, *Synthesis*, 2010, **2010**, 2609–2615.

[5] C. Hoyer, P. Schwerk, L. Suntrup, J. Beerhues, M. Nössler, U. Albold, J. Dernedde, K. Tedin and B. Sarkar, *Eur. J. Inorg. Chem.*, 2021, **2021**, 1373–1382.

[6] J. Beerhues, H. Aberhan, T.-N. Streit and B. Sarkar, *Organometallics*, 2020, **39**, 4557–4564.

[7] L. S. Lloyd, A. Asghar, M. J. Burns, A. Charlton, S. Coombes, M. J. Cowley, G. J. Dear, S. B. Duckett, G. R. Genov, G. G. R. Green, L. A. R. Highton, A. J. J. Hooper, M. Khan, I. G. Khazal, R. J. Lewis, R. E. Mewis, A. D. Roberts and A. J. Ruddlesden, *Catal. Sci. Technol.*, 2014, **4**, 3544–3554.
